# Let’s Talk About Potentially Modifiable Risk Factors for Dementia

**DOI:** 10.1192/j.eurpsy.2025.1729

**Published:** 2025-08-26

**Authors:** M. B. Fonseca, A. S. Pires, S. Mouta, D. Figueiredo, M. Pires, I. Soares

**Affiliations:** 1 Psychiatry and Mental Health, ULS Guarda, Guarda, Portugal

## Abstract

**Introduction:**

Dementia is a syndrome usually chronic and progressive, in which cognitive deterioration occurs at a rate greater than what is expected due to natural aging. (WHO). Because it has a major impact on functional levels, dementia is a major cause of dependence and loss of autonomy in the elderly population.

As the population ages, the prevalence of dementia disorders is expected to increase over the years. This will lead to serious problems in the healthcare sector and social welfare. As populations age, the prevalence of dementia will increase, with the number of people with dementia worldwide expected to rise to 150 million by 2050.

This group of neurocognitive disorders has multiple possible aetiologies, constituting a highly heterogeneous group. However, several studies within this area have been searching for and studying modifiable risk factors, to predict and reduce its incidence.

**Objectives:**

The authors propose a review of the various modifiable risk factors involved in developing dementia syndromes.

**Methods:**

Review of the existing literature about modifiable risk factors for dementia, using the keywords: dementia, modifiable risk factors, prevention. The results were selected taking into account the degree of relevance.

**Results:**

Several studies argue that several modifiable risk factors may be involved in the development of dementia, from early to late life, such as less education, mental illnesses such as depression, hearing, and vision loss, high LDL cholesterol, and social isolation, among others.

**Conclusions:**

Dementia has a significant mortality rate and is responsible for great disability and dependence in older populations. The implementation of global health measures, focusing on prevention and reducing risk factors, could be an important link in reducing the prevalence of dementia in the future.

**Disclosure of Interest:**

None Declared

